# Observations of community-based multidisciplinary team meetings in health and social care for older people with long term conditions in England

**DOI:** 10.1186/s12913-022-07971-x

**Published:** 2022-06-08

**Authors:** Nick Douglas, Nicholas Mays, Mustafa Al-Haboubi, Tommaso Manacorda, Lavanya Thana, Gerald Wistow, Mary Alison Durand

**Affiliations:** 1 Policy Innovation and Evaluation Research Unit (PIRU), Department of Health Services Research and Policy, London School of Hygiene & Tropical Medicine, London, UK; 2 Now School of Psychology, University of Sussex, Brighton, East Sussex, UK; 3 Now Public Health, Advocacy and Welfare, Italian Multiple Sclerosis Society, Genoa, Italy; 4 Care Policy & Evaluation Centre, London School of Economics & Political Science, London, UK

**Keywords:** Multi-disciplinary teams; health and social care integration; non-participant observation

## Abstract

**Background:**

Community-based multi-disciplinary teams (MDTs) are the most common means to encourage health and social care service integration in England yet are rarely studied or directly observed. This paper reports on two rounds of non-participant observations of community-based multi-disciplinary team (MDT) meetings in two localities, as part of an evaluation of the Integrated Care and Support Pioneers Programme. We sought to understand how MDT meetings coordinate care and identify their ‘added value’ over bilateral discussions.

**Methods:**

Two rounds of structured non-participant observations of 11 MDTs (28 meetings) in an inner city and mixed urban–rural area in England (June 2019-February 2020), using a group analysis approach.

**Results:**

Despite diverse settings, attendance and caseloads, MDTs adopted similar processes of case management: presentation; information seeking/sharing; narrative construction; solution seeking; decision-making and task allocation. Patient-centredness was evident but scope to strengthen ‘patient-voice’ exists. MDTs were hampered by information governance rules and lack of interoperability between patient databases. Meetings were characterised by mutual respect and collegiality with little challenge. Decision-making appeared non-hierarchical, often involving dyads or triads of professionals. ‘Added value’ lay in: rapid patient information sharing; better understanding of contributing agencies’ services; planning strategies for patients that providers had struggled to find the right way to engage satisfactorily; and managing risk and providing mutual support in stressful cases.

**Conclusions:**

More attention needs to be given to removing barriers to information sharing, creating scope for constructive challenge between staff and deciding when to remove cases from the caseload.

**Supplementary Information:**

The online version contains supplementary material available at 10.1186/s12913-022-07971-x.

## Background

A key driver for the integration of health and social care (H&SC) is that many (older) people live with multiple chronic conditions and require the support of both health and social care services [1]. The need for better coordination of H&SC in the UK has been noted repeatedly but progress is slow [2]. Multidisciplinary teams (MDTs) have been found to be systemically important interventions contributing to health and social care integration in the UK [3]. While numerous studies have focussed on MDTs working in various healthcare contexts, (e.g. cancer, primary care, mental health, chronic obstructive pulmonary disease and other chronic diseases) [4–8], there is less research on MDTs incorporating *both* H&SC professionals [9]. This is despite studies suggesting that involving social workers in healthcare provision can have positive effects on health outcomes and reduce costs [10, 11]. A national study evaluating H&SC integration in England presented an opportunity to address this research gap by exploring the functioning of MDT meetings involving a range of health and social care professionals in community-based settings.

In late 2013, the then Department of Health in England initiated the Integrated Care and Support Pioneers Programme [12]. Fourteen geographically and socio-demographically diverse areas of the country, expanded to 25 in 2015, were selected to drive health and social care integration ‘at scale and pace, from which the rest of the country can benefit’. They were expected to deliver patient-centred care, improve patient experience and improve financial efficiency through drives toward whole system integration between health, social care, the voluntary sector and other public services [13]. An initial process evaluation found that community-based, integrated H&SC, multi-disciplinary teams (MDTs) constituted the most commonly reported, systemically important integration intervention across the first 14 Pioneers [51], and a 2019 key informant survey of all 25 confirmed this still to be the case [52].

All Pioneers that had reported deploying community-based MDTs as integral to their local service integration strategies were approached to participate in an in-depth economic and impact evaluation of their MDTs within the wider evaluation [51]. After discussions with 10 sites that volunteered, it became apparent that only two Pioneers had MDTs that routinely involved health and social services staff, and were willing and able to facilitate the MDT sub-study. We conducted two rounds of non-participant observations [14] of H&SC MDT meetings in these two Pioneers.

The Covid-19 pandemic has further starkly highlighted the limitations of disjointed systems of care and reinforced the vital importance of H&SC integration [15], underscored by the Government’s recent White Paper on health and social care integration [16]. In-depth study of the functioning of community-based, H&SC MDTs offered an important window into integration initiatives operating at the level of local health and social care economies.

## Methods

Our research questions were: how do MDT meeting participants work to perform coordinated, integrated, person-centred care for those on their caseload (as envisaged in the National Voices ‘I Statements’ that informed the definition of patient-centredness for the Pioneers programme [17]); and what are the observable decision-making processes?

**Fig. 1:**
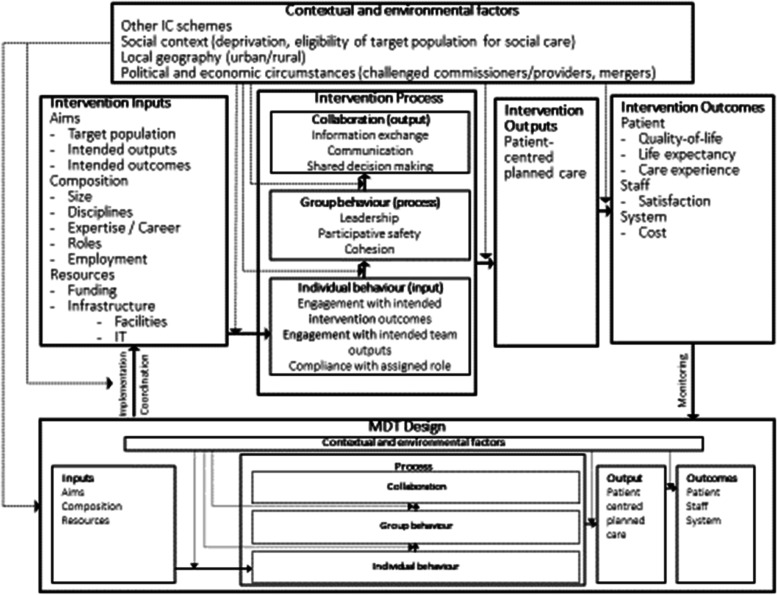
Conceptual Model of Community-Based MDT Functioning

We developed a conceptual model of community-based MDT functioning based on a rapid review of theories and studies of multidisciplinary team working in the UK. Much of the research and conceptualisation related to healthcare settings (sometimes hospital based), with teams not including social care staff [e.g. 18, 19–21]. However, one study in England examined features of team functioning in the chronic disease treatment setting that included social care staff and which was instructive for our study [8]. With some adjustment, derived from the Pioneer logic models developed in the early evaluation of the programme [51], this became the basis for the conceptual model guiding data collection and analysis in the current study (see Fig. 1).

The conceptual model guided the development of the data collection tools which covered:

- Logistics of the meeting (e.g., meeting space, administrative support): extent to which this facilitated/hindered interaction etc.
- Health and social care resources: extent to which the team was facilitated/hindered by the availability of resources to plan care and meet patients and carers needs. (N.B. beneficiaries were identified by a range of terms such as patient, client, service user etc.,—we have used the term ‘patient’ here for consistency.)
- Mechanisms for data sharing: ways in which information about patients was shared and extent to which this facilitated/hindered interaction.
- Leadership/Chairing: extent to which the agenda and depth of discussion was set and managed by one (or more) participant(s) whose role was acknowledged by others.
- Cohesion/participative safety/conflict resolution: extent to which discussion was characterised by consensus building, respectful interaction or conflicting views or positions, and how differences of view were reconciled.
- Patient-centeredness: extent to which patients’ and/or their informal carers’ preferences and priorities were explicitly discussed.
- Information exchange/shared decision making: extent to which discussion was characterised by collegial information sharing and decision-making.
- Reflexivity: extent to which decisions were reflected on.

Thus, we sought to: 1) describe the mix of professionals present at meetings and any apparent gaps in expertise; 2) assess key aspects of team functioning and interaction; 3) describe any observable logistical facilitators and/or barriers to the effective operation of meetings; and 4) understand the nature of decision-making during meetings. We additionally sought to identify the possible ‘added value’ of integrated meetings (over bilateral discussions between professionals).

### Inclusion Criteria

H&SC integrated community-based MDTs involved in the study had to: 1) include both primary and social care, as well as allied healthcare professionals, and in some instances, the community and voluntary sector (CVS); 2) bring these professionals together in a shared process of care coordination; and 3) have a target caseload that included people aged 55 and over with multiple long-term conditions, i.e. those who often need complex care coordination and high levels of health and social care support [22, 23]. We included people over 55 years on the grounds that the proportion of the population with more than one long-term condition rises appreciably from that age onwards [24, 25].

### Settings and Context

MDTs were located in two Pioneer sites. Pioneer 1 (an inner-city area in England) hosted 8 MDTs coordinated by NHS administrators, which met across a two-week cycle of 13 scheduled meetings. Pioneer 2 (a mixed urban–rural area in England) hosted 2 MDTs, individually administered by GP practice staff and 1 MDT, which was administered by clinical staff (nurses, physiotherapists and occupational therapists) located in an NHS acute hospital trust. Two of these MDTs met weekly while the third met daily on weekdays.

### Data Collection

We observed 28 meetings in two rounds of observations (June 2019; December 2019 to February 2020) involving all 11 MDTs in two Pioneer localities participating in the in-depth part of the evaluation. All the meetings took place in NHS facilities (mostly primary care), the quality of which varied considerably, from bright, modern multi-purpose clinics to aging GP surgeries. In Pioneer 1, meeting rooms were often cramped, with insufficient seating, tables, or clear lines of vision for participant face-to-face interaction.

MDT attendees were provided with an information sheet and consent form in advance, which explained the right to withdraw consent and was signed at the beginning of each meeting observed. Information was posted on meeting room doors informing latecomers about observations taking place. Where attendees notified administrators in advance that they did not wish to participate, we did not attend. One staff member declined to participate in two meetings in round one but these MDTs’ meetings were observed in round two.

Each meeting was attended by two researchers from the team of six, with the exception of two round 2 meetings at Pioneer 2, where one researcher attended. Meetings lasted on average over two hours (range: approximately 45 min to three hours).

As it was not possible to obtain informed consent from the patients being discussed at the MDT meetings, our research ethics approvals did not permit us to audio record meetings or note any patient identifiable information, and such information was obscured from researchers during meetings. Thus we observed and noted the process of the meetings, using structured, non-participant observation but not the decisions related to specific patients [14, 26].

To record data in a systematic and rigorous way in complex, fast-moving meetings, we employed structured observation pro formas, grounded in our conceptual model. For round 1, the pro forma focused on capturing process-orientated data related to the dimensions of team functioning in meetings, as identified above. In round 2, the pro-forma focused on MDT decision-making processes (see Supplementary Table 1). Following observations, researcher pairs conducted immediate post-meeting discussions, reviewing together the broad content of data recorded, and field notes were written shortly thereafter.

### Analysis

We decided to analyse the data as a research group since it comprised structured notes and reflections made by researchers rather than direct recordings of MDT meeting discussions. We conducted two group analysis sessions, lasting five hours in total (facilitated by ND). The research team was diverse in terms of gender, ethnicity, discipline, seniority and institution. Although all non-clinical health services researchers, the team had considerable experience of observing consultations and clinical procedures in a range of settings. As field observation invariably involves researcher interpretation of events, it was important as part of a reflexive approach to be able to interrogate each other’s perceptions and interpretations to develop a shared understanding of the MDT meetings observed.

Before group analysis sessions, fieldnotes were shared and team members prepared responses, based on their own fieldnotes, to a series of analytical questions related to the key themes of meeting context, process, team functioning and decision-making. During the sessions, each researcher: 1) provided general, unstructured feedback about their observations; 2) gave structured feedback in relation to the analytical questions, followed by open group discussion; 3) identified the most salient findings for reporting; and 4) identified further research questions for additional data collection and analysis. This allowed us to present, compare, contrast and ‘test’ interpretations and insights through an interrogative group process. A seventh researcher who had not participated in the observations acted as a ‘critical friend’ [27]; asking questions, challenging and offering alternative explanations to test theories and observations.

The first group analysis meeting identified that MDT decision-making required further data collection and we focussed largely, but not exclusively, on this topic in our second round of observations and analysis meeting.

The two group analysis sessions were audio recorded, transcribed verbatim and transcripts treated as primary data of equal standing with fieldnotes (researcher speech is used to illustrate key findings). Group analysis transcripts reflected robust discussions, which included ‘testing’ of individual interpretations of events observed and noted in situ during the MDT meetings, which had initially been discussed in pairs immediately post-observation and noted.

A thematic analysis was undertaken of the transcripts, using a six-stage process: data familiarisation, generating initial codes, searching for themes, defining and naming themes, reporting the data [28]. ND deductively coded the transcripts using the dimensions of team functioning and decision-making in our MDT model (see Fig. 1) and analysed them thematically. MAH reviewed a sample of the coding (approximately 25% of codes). No significant discrepancies were identified. The wider team reviewed and commented on the developing analyses. NVivo 11 Plus was used to manage the data [29].

## Results

In the following, we explore key dimensions of team functioning from our conceptual model, particularly those related to meeting inputs and processes. We then explore data on whether bringing groups of health and social care professionals together in this way appeared to offer additional benefits for coordinating and delivering care.

### Inputs

#### Matching Attendees and Case Needs

Some staff groupings tended to be present at all meetings: administrators, GPs, social workers, (including managers), and nurses, (including Clinical Nurse Specialists and Community Matrons). However, a sub-set of Pioneer 2 meetings differed (attended by nurses, occupational therapists and physiotherapists, administered on a rotating basis). Mental health workers were present at Pioneer 1 meetings. CVS attendees were present in Pioneer 1 and 2 meetings. Where GPs attended, numbers varied. In most meetings, they remained throughout, in others leaving after case presentation. Administrators (where present) sometimes contributed in depth to meeting discussions.

Attendance by professional groupings appeared partly to be a product of caseload profiles. MDTs in both sites sought to prevent hospital (re)admission or manage post-hospital discharge, and barring one, were primarily managed from GP practices but cases discussed during meetings at the Pioneer 2 sites seemed to be older people, with fewer problems related to housing, substance misuse or learning disability. Despite the fact that attendance appeared to be shaped by the types of cases to be discussed, sometimes key staff were not present whose expertise matched the requirements of the cases: for example, none of the meetings had direct input from local authority housing services and this was particularly notable in the Pioneer 1 meetings, where chronic housing problems were often discussed. The frequent absence of a representative from the local NHS acute trust at Pioneer 1 meetings was commented on by one GP. Learning disability services and substance misuse services were not represented at any of the meetings, which may have been helpful. When two substance misuse workers attended simply to introduce their service at a meeting in Pioneer 1, one became involved in productive discussions about patients, demonstrating the value of being able to access a wider range of expertise.

*“[The drug and alcohol worker] immediately jumped in to say, ‘Oh, I know that patient,’ or, ‘You could refer that patient to me.’ So, they were immediately acting as a potential resource for the team, which seemed interesting, and I think the staff that were there found it really helpful.” (Researcher 1, Team Analysis Meeting 1).*

#### Sharing Intelligence

Access to the internet, IT hardware (PCs, laptops, tablets) and software (databases containing information about patients and their carers) appeared to be critically important in meetings, given the functions of the MDTs. In Pioneer 2 meetings, designated administrators and clinical staff primarily accessed the relevant databases. In the Pioneer 1 sites, in addition to primary care databases, social services databases, were heavily relied upon due to the nature of patients’ needs or service use. These databases often appeared to contain the most detailed information about patients and their carers but were only accessible to the social workers. We observed that attendees from different organisations often could not access each other’s systems (either for technical or information governance reasons): the social services, CVS and mental health databases in Pioneer 1 were entirely separate and required workers from these organisations to be present to access them. There was one highly inefficient system that prevented senior nurses from accessing two required databases on the same device at once. Also, visiting staff had to bring their own equipment, which was variable in quality, whereas GPs often used PCs onsite.

Reliable access was also often variable, with examples of difficulty logging on to systems or slow/unreliable connections, particularly in Pioneer 1. We observed that access to information was determined to some extent by the quality of the IT connections and/or hardware available. Accessing and sharing information from different systems—a key function of the meetings – appeared to consume an inordinate amount of time, especially in the Pioneer 1 meetings.

*“There were so many different systems that they were trying to access in the meetings and they still didn’t have access to all the ones they needed.” (Researcher 3, Team Analysis Meeting 1).*

### Meeting Processes

#### Leadership and Chairing

Meetings varied in the extent to which explicit chairing was observable. In Pioneer 2 and some of the Pioneer 1 meetings, formal chairing, often performed by GPs, nurses or sometimes social workers, was evident. In other Pioneer 1 meetings, chairing was more informally shared between two or more attendees (most usually a GP, social worker and/or senior nurse).

*“At the [name removed] one that we attended, where the social worker was chairing, it was a very obvious chairing, […] He’d bring it to a point where he’d say, ‘Okay, so what are the actions?’ and that tended to focus minds. […] It wasn’t quite as formal in the other two meetings that we attended, and in fact, after one meeting [we researchers] discussed who was the chair there.” (Researcher 1, Team Analysis Meeting 1).*

#### Cohesion, Participative Safety and Conflict Resolution

Meetings appeared to be characterised by uniformly collegial, professional and respectful interactions. We observed humorous exchanges and friendly banter. Attendees were on first-name terms and interacted in ways that suggested established working relationships. An exception was a meeting where the atmosphere was perceived by both researchers to be tense.

We observed that some CVS attendees in the Pioneer 1 meetings contributed infrequently to discussions, although the reasons for this were unclear. We did not observe lesser participation by particular groups in any of the Pioneer 2 meetings, although certain professionals appeared to be more vocal or more involved in chairing, as described. We observed no major distinctions of status or hierarchies affecting meeting dynamics.

Interactions were collegial and largely consensus-based, which raised questions for us about the extent of challenge in the meetings. For example, we saw no overt examples of conflict. However, in instances where a degree of challenge did take place, this appeared to add to the quality of the discussion.

*“In one of the meetings I attended there was a mental health worker that just asked questions, like continually challenging the discussion and the debate that was happening, which was really helpful because it really unpicked what was going on.” (Researcher 6, Team Analysis Meeting 2).*

#### Sense-Making

We also paid attention to the deliberation processes in meetings, and were able to identify a consistent pattern of:

- Case presentation – an attendee introduced the patient, giving salient facts and reasons for referral or addition to/presence on, the caseload.
- Information seeking/sharing – attendees searched their individual databases or drew on personal knowledge to identify what was known about the patient and any informal carers. This was a major element of the process.
- Narrative construction – attendees shared information to create a historical reconstruction and collective view of the patient’s current needs, challenges, or situation. This included information such as demographics, health and care needs and problems, living and family circumstances, service usage, formal and informal care and support being provided, barriers and facilitators to working with the patient, and likely acceptability of potential service/treatment options to patients and carers.
- Solution seeking – suggestions and proposals to address the needs identified were considered, often involving sharing information about each other’s services and other local services that might be appropriate.
- Decision-making and task allocation – actions were agreed and allocated, including further assessments and referrals, whether to remove the patient from the caseload or review at a later date.

This was not always a linear process, especially if the patient was already on the caseload, where there was less focus on narrative construction and more on ‘status update’. The patient databases were especially important in establishing the narrative and what actions had already taken place outside the meeting.

#### Pace and Depth

Time spent discussing cases varied considerably. In Pioneer 1 meetings in particular, extensive deliberations were observed (though cases were often also discussed rapidly). However, in one MDT in Pioneer 2, case discussion was very rapid, to the extent that following the substance of deliberations was often challenging. In Pioneer 1, we observed that participants sometimes struggled to discuss all intended cases in the time allotted, with deliberations becoming more hurried towards the end of meetings.

#### Who Decides?

Making care co-ordination decisions was central, principally about whether to retain or discharge a patient from the caseload and, if retained, necessary actions to provide appropriate care and support. Criteria for adding a particular patient to the caseload or, in many cases, for discharging them, were not readily transparent, though this may be related to our partial view of administrative processes.

Where decisions were clear, we frequently observed decision-making by key dyads and triads, which varied in composition depending on professional groups present and the nature of the case. GPs, social workers and nurses were frequently central to these dyads/triads but other configurations were seen, particularly in Pioneer 2, where GPs were not present and nurse-physiotherapist-occupational therapist configurations were observed to be making care-related decisions.

Some deliberations were extremely rapid: on receiving some information from an attendee, the professional(s) from the service(s) best placed to act or meet the identified need, made a swift decision about patient management actions and the discussion moved promptly to the next case. In other cases, there was lengthy and detailed deliberation. In some of the Pioneer 1 meetings, perhaps due to less structured chairing, responsibility for moving the discussion to a conclusion was sometimes unclear.

*“It sometimes wasn’t clear that a decision had been reached or indeed precisely what the decision was. There was quite a lot of deferral and not much discharge, although that is different in different places.” (Researcher 3, Team Analysis Meeting 2).*

However, in other Pioneer 1 meetings, GPs appeared to determine when a discussion was concluded, possibly because they had ongoing, ultimate responsibility for the patient, while other agencies were involved only if they had a relevant service to offer and the patient was eligible (e.g., CVS, mental health services, social services).

*“While other organisations’ representatives somehow are used to the fact that if they cannot do anything for the patient, it’s not their job, it’s always the GP’s job to some extent.” (Researcher 5, Team Analysis Meeting 2).*

#### Deferring Difficulty

Requests for further assessments and tests, especially for new or returning patients, could be an important precursor to decision-making, but we also observed instances where further assessment appeared to be used to defer difficult decisions in cases where the course of action was unclear.

*“The sorts of actions they committed to, most of them, I don’t want to use the term ‘kicking the can down the road,’ but it was requesting additional assessments or more information.” (Researcher 2, Team Analysis Meeting 1).*

Differences in the caseloads between the two sites may have been significant in decisions to defer. In Pioneer 2, the caseload was predominantly frail people, over 75 with multiple complex health conditions and related social care needs. This group was also prevalent in Pioneer 1 meetings’ caseloads. However, their caseloads also included more younger people and those whose problems included homelessness, substance misuse, mental illness and learning disability. Here, we sometimes observed professionals struggling with what appeared to be highly complex and at times, distressing cases, where problems were severe and enduring, multiple options for intervention had been exhausted and services had difficulty engaging with patients.

*“Almost all of them [patients] […] had multiple, interrelated, complex life difficulties that didn’t neatly fit into any known category. What I thought I witnessed was a combination of people trying to, as you say, puzzle out, ‘What could we usefully do?’ and, ‘Is there a limit to what we should be expected to do?’” (Researcher 4, Team Analysis Meeting 1).*

We questioned whether it was always clear in what circumstances MDTs could reasonably decide to withdraw from case management and reallocate cases that had apparently exhausted the MDT process.

*“What they did in those sorts of cases was they kept them on the caseload because they didn’t know what else to do, and it seems to me that there needs to be some sort of discussion about how are they given permission to say, ‘We’ve run out of road here,’ and ‘What’s the [next] response to those patients?’.” (Researcher 3, Team Analysis Meeting 1).*

#### Patient-Centredness

We explored whether patient-centredness was evident as part of the deliberations. The CVS organisation present in the Pioneer 1 meetings attended as a service provider but it was unclear whether it also performed a formal patient advocacy function. Although patients were never present, we noted examples of substantial patient-centredness in the discussions, both explicitly, in considering needs and preferences, and implicitly, through the process of constructing the patient’s narrative.*“To what extent was patient-centredness a feature of the meetings? To a very large extent. I felt that whenever they were proposing something, they were immediately thinking about whether this would be suitable for that patient, whether it would work, so it was an integral part of the conversations.” (Researcher 2, Team Analysis Meeting 1).*

### Added Value?

We explored the issue of whether there was ‘added value’ from bringing together professionals in MDT meetings, further to what might have been achieved by them working separately. The meetings appeared to offer an effective alternative way to share information about patients.

*“These meetings provide a forum or a setting in which you could have discussions that wouldn’t be appropriate to have over an email, for example, discussing about a case and someone giving them the background, which is not available on any of their systems. I felt that was a definite added value that would make them decide or reach decisions they wouldn’t have otherwise, knowing the additional background.” (Researcher 2, Team Analysis Meeting 2).*

The ability to consult databases and give ‘real-time’ updates to inform decision-making was another observed benefit.

*“One thing I would add to the added value question is the immediacy of what was happening in the room, which they wouldn’t have got if they phoned or emailed. They could get an immediate response and update, which was often, I felt, really beneficial to patient care.” (Researcher 6, Team Analysis Meeting 2).*

Mutual understanding of decisions made by individual services regarding planning or patient care was enhanced by having attendees from different services and organisations present.

*“They were contrasting the patient-centred narrative with their experience of the services, trying to identify what misfired in the encounter between the need and the potential supply, […] [asking] ‘Why do we have a note saying […] that they didn’t meet the criteria?’” (Researcher 5, Team Analysis Meeting 2).*

Also, for patients that services had difficulty engaging, or cases that were difficult to resolve, attendees could discuss who had the best rapport and likelihood of engaging them to achieve potential solutions.

*“Another instance [of added value] was a discussion about who should approach the patients to get them to do something that they are not keen to do. […] Everyone felt it was in the patient’s best interests but who has the best rapport with the patient to do that? I can’t imagine them exchanging emails saying, ‘You ask…” (Researcher 3, Team Analysis Meeting 2).*

Another form of added value for participating services was shared identification and management of risk in often complex cases and uncertain circumstances.

*“In part, the decision making relates to that, […] what they’re concerned about as well are things like the risks, ‘If we don’t do X, Y might happen.’” (Researcher 1, Team Analysis Meeting 2).*

Sharing clinical learning or information about how organisations and the local H&SC economies functioned, and how to navigate this was also a beneficial consequence of MDT meetings.

*“Different organisations explaining to others how they operate was a definite added value, and occasionally the medical background that the GP was explaining, which helped other organisations understand why they need to provide certain support, that was a definite added value.” (Researcher 6, Team Analysis Meeting 2).*

However, we noted scope to enhance shared learning by making more space for reflexivity within meetings. For example, Pioneer 1 meetings had a standing agenda item to discuss what had been learned but this was often overlooked or dealt with in a perfunctory way at the end of meetings. However, when shared learning occurred, it could reportedly have benefits beyond the immediate MDT meeting.

*“[A GP] said to us afterwards that one of the key components for her, I guess in terms of added value, was that she was learning what was available and what people could contribute […] and that she would take that learning back to her own practice.” (Researcher 6, Team Analysis Meeting 2).*

The format of the MDT meetings also appeared to enable participants to support one another practically and emotionally.

*“They weren’t working in isolation. They were able to bring and receive support, probably also emotional support for dealing with quite difficult, in some cases, patients with really complex mental health and social needs, as well as physical.” (Researcher 6, Team Analysis Meeting 2).*

## Discussion

Our study sought to understand how MDT meeting participants work to perform coordinated, integrated, person-centred care for those on their caseload. Our conceptual model of MDT functioning suggested that we might most usefully focus upon key inputs, meeting processes and observable ‘added value’ of MDT meetings. We were able to explore: the fit between professional groups attending and needs identified in cases, intelligence sharing, leadership and chairing, participation, sense-making and decision-making, patient-centredness and potential added benefits of bringing groups of professionals together in this way for care coordination and delivery.

We observed that meeting spaces were sometimes unsuitable. A central component of MDT working is adequate physical facilities to support the work [30] and the quality of working space has been found to be a factor in interprofessional communication and collaboration in healthcare [18, 31]. Austerity funding has been linked to the significant deterioration of many NHS facilities [32]. Therefore, proper resourcing [9] and investment in infrastructure may be necessary where facilities are not fit for purpose.

We identified that teams primarily consisted of administrators, primary care staff, allied healthcare professionals and social workers, with some CVS staff and mental health staff. It is unclear whether this simply reflected local caseloads, the availability of different professionals in different localities or a deliberate decision. It is also unclear what the optimum composition of MDTs is but local flexibility and responsiveness to context is likely to be needed [8, 33, 34]. In common with other studies, we were able to identify staffing gaps in some MDTs, such as specialists in substance misuse, housing and learning disability [9]. It was beyond the scope of the present study to examine whether this was related to wider structural factors in the H&SC economies within which the MDTs were located (e.g. lack of resourcing, difficulties recruiting specialists to the MDTs or more broadly) and we can only pose the question here. However, Raine, et al. [8] found that wider structural factors can impact on the performance of MDTs, reporting that MDT decisions were less likely to implemented in more deprived areas. Future research might usefully focus on whether better outcomes might be achieved by identifying ‘core’ (always needed) and ‘occasional’ (sometimes needed on an ad hoc basis) staff, according to the prevailing case mix. Raine, et al. [8] also reported a lack of administrative support, which negatively impacted on ability to formulate treatment plans in some MDTs studied. This contrasted with our observations in this study that administrative support was available and well-integrated into team meetings and planning.

Our study was novel in exploring the day-to-day impact of IT systems for team functioning, which has been under-explored [8, 19]. MDTs were hampered by obstructive information governance, lack of interoperability between required patient databases and inconsistent IT access. Yet sharing information, supported by efficient information systems and digital interoperability, are essential for effective functioning of MDTs and H&SC integration [9, 35–37]. Teams had found workarounds but this required professionals to bring their own solutions (most often in the form of a laptop). Until such barriers are removed, as was promised in the Government’s 2018 strategy on technology in H&SC in England [38], MDTs can be expected to continue to spend a large portion of their face-to-face deliberations simply collating information from disconnected clinical and managerial databases.

Other studies have identified the importance of clear leadership to effective team functioning [6, 9, 18, 39, 40]. Our study focussed more specifically on meeting chairing, which we found was variable in the MDT meetings observed. Further work might usefully focus on the specific styles and skills needed for effective MDT chairing where ‘distributed leadership’ [41] may be most appropriate.

In terms of processes of interaction, unlike others, we did not observe medical dominance or evidence of explicit status differences between professionals [8, 9], although there was deference to GP views as to when discussions should be concluded in some Pioneer 1 meetings. We did note less vocal participation by some CVS attendees in Pioneer 1. This warrants further investigation as it may have implications for the local sensitivity of services to need and the ability of the statutory sector to make the best of the local CVS. Overall, we found that meetings were characterised by professional respect, cohesion and collegiality, which have all been described as important to effective MDT working [18, 42]. One US study suggests that collaborative care practice may potentially reduce physician ‘burnout’ [43].

Decision-making was largely consensus-based with core dyads and triads of professionals involved. However, chairing and decision-making might have been clearer at times, particularly related to discharge from the caseload and deciding when cases had exhausted the MDT process. The high degree of cohesion did raise questions about the ability of professionals sufficiently to challenge one another, which has also been identified as a core function of effective team working [44].

Significantly for understanding MDT processes, we were able to identify the typical sequence for case deliberation: case presentation, information seeking/sharing, narrative construction, solution seeking, decision-making and task allocation. We found evidence of patient-centredness in this process, though there was potential scope to strengthen ‘patient-voice’ and challenge in the process. Raine, et al. [8] argue that MDT decisions that take account of patient preferences are more likely to be implemented. Studies from the Netherlands shed light on how patients might be more directly involved in integrated team meetings. A tailored approach, based on trust and equality, prior information and preparation, with a professional designated to maintain ongoing communication before, during and after meetings, removing barriers such as jargon and taking account of the patient’s ability and preferences regarding participation were found to be important factors in effective patient involvement [45, 46].

In comparing and contrasting the MDT meetings observed, the major finding is the considerable diversity in: the fitness (or not) of meeting facilities and IT; the patient caseload profile; the professional groups attending; chairing and administration; and the pace of meetings. Where there was perhaps more similarity was in the intended outcomes (avoidance of hospital admission and discharge management), deliberation processes and the primacy of decision-making dyads and triads.

On the overall question of ‘added value’ of the MDTs, we found indications of this in a number of respects: sharing information about patients, often in ‘real-time’; learning about services, processes and decision-making of other participating agencies; planning strategies for patients and/or their carers that services found difficult to engage; managing risk and; sharing support for often distressing and stressful cases.

### Strengths and Limitations

MDTs are often proposed as a solution to provision of more integrated services, yet these are infrequently studied and even less so, directly observed. There were a number of strengths to this study. Our study fits with calls for research that is grounded in the practice and processes of how teams interact [9, 47]. Our conceptual model of MDT functioning also enabled us to overcome a significant problem regarding the lack of shared identification and naming of key constructs in team functioning [48]. It helped us identify key factors of interest, focus the observations and inform research instrument development. We also found that our model was empirically supported by observational data, in that key concepts identified as potentially important to team functioning were evident in the meetings that we observed (though dimensions relating to outputs and outcomes remain open to further inquiry). Our model may be useful for other studies, particularly those investigating team processes.

Also, our two-step approach of immediate, post meeting review, subsequent group analysis, and treating the notes of structured observations of meetings as primary data, had a number of advantages. This facilitated a careful and reflexive approach to sharing, ‘testing’ and sifting the data through a process of review and discussion. It enabled us to address issues of inter-observer variability, checking insights and accounts while still ‘fresh’ and after a period of reflection as part of a considered group process, thus enhancing study rigour. The diversity within the research team and deploying a researcher who had not participated in the observations as a ‘critical friend’ [27] further strengthened this.

In the most fast-moving meetings, it would have been preferable to audio record had this been permitted. We were also unable to record or analyse information about the process by which patients entered the MDT caseload or the clinical and social care decisions that the MDTs made (only their case management processes). This potentially would have aided a fuller understanding of the organisational/administrative work that went into preparation and follow up for each meeting. We also considered using validated instruments to observe MDT meetings (see Rossell et al., for examples of the Meeting Observational Tool (MDT-MOT) and Metric of Decision-Making (MDT-MODe) being used) [49]. However, our scoping work indicated that existing tools were developed for use in very different clinical settings, e.g., cancer treatment, with exclusively medical teams and not applicable without modification to the multi-disciplinary, community-based context in which we were working. As we sought to conduct a more exploratory study, qualitative methods were also preferable. However, the development of such tools for these settings may potentially advance the field of study. Another possible limitation was a social desirability ‘Hawthorne effect’ [50], which may have led attendees to modify their behaviour to minimise conflict and challenge, though our post-meeting reviews with administrators suggested that the meetings we observed were typical. The variation in the MDTs observed was useful to the study: we included MDTs in two diverse areas with very different geographic and socioeconomic contexts but the findings were very similar, suggesting that they were not idiosyncratic. However, a larger scale study would be useful to further explore transferability to wider contexts.

## Conclusions

This analysis has primarily focussed on some of the key process dimensions of MDT meetings and has generated insights into staff interactions in case management and how they conduct activity between themselves, which are generally hidden from view. The study was aided by the variation of MDTs in terms of their physical environments, team composition, frequency of meetings and the populations and geographical locations they served, which may have enabled a diverse range of MDT processes to be observed. As such, it is a significant contribution to understandings of day-to-day operational practice in MDTs within Integration Pioneer localities. Other aspects of the roles they played in service integration, including whether different MDT models produce different outcomes for patients and carers are the focus of our wider evaluation of the Pioneer programme.

## Supplementary Information

**Additional file1:** **Supplementary Table 1**

## Data Availability

The datasets generated and analysed during the current study are not publicly available because they contain information that would identify the research sites, individuals, and/or case-material and it would not be feasible to redact or otherwise anonymise them. The data custodian is Professor Nicholas Mays, London School of Hygiene and Tropical Medicine. All methods were carried out in accordance with the Research Governance Framework for Health and Social Care (2005) and the UK Policy Framework for Health and Social Care Research (2021). In addition, the research complied with regulatory requirements contractually specified for studies funded by the Department of Health and Social Care.

## Abbreviations

CVS: Community and Voluntary Sector
GP: General Practitioner
H&SC: Health and Social Care
IT: Information Technology
MDT: Multi-disciplinary Team
NHS: National Health Service
PC: Personal Computer
