# Supplementary material for: Observations of community-based multidisciplinary team meetings in health and social care for older people with long term conditions in England
Source: BMC Health Serv Res. 2022 Jun 8;22:758. doi: 10.1186/s12913-022-07971-x (PMC9175164; doi:10.1186/s12913-022-07971-x)
Supplement: Supplementary file 1 — Additional file1: Supplementary Table 1 [file 12913_2022_7971_MOESM1_ESM.docx]

**Supplementary Table 1**

| **Data Gathering** |  |
| --- | --- |
| Round 1: Components of Process Pro-forma | Round 2: Components of Decisions Pro-Forma |
| - Instructions on completion. - Definitions of key dimensions of team functioning that were of interest (informed by our developing model of MDT functioning). See Figure 1. - Part 1 – A record of the researchers present, date, time and location of the meeting, start and finish time, list of MDT members present and organisations represented at the meeting. - Part 2 – A series of structured tables to record notes taken in situ. A number was ascribed to each case as it was discussed. The forms recorded: 1) significant key events, behaviour, conversation etc observed between MDT members and further detailed notes about these, 2) a pre-identified code as to which of the key dimensions of team functioning the observations related to. - Part 3 – A series of tables completed after the meeting to gather reflections on the meeting, organised around the key dimensions of team functioning of interest and any other emerging relevant issues. | - Part 1 – A record of the researchers present, date, time and location of the meeting, start and finish time, list of MDT members present and organisations represented at the meeting. - Part 2 -   - Agency making referral.   - Professional introducing case.   - Professional(s) contributing.   - Missing agencies.   - General issues and problems team sought to address.   - Decisions reached (e.g. retain, discharge, refer).   - Decision makers.   - Referrals agreed. - Part 3 - Completed post hoc, with meeting administrator.   - Total number of cases discussed.   - How patient lists were compiled.   - Typicality of the meeting in terms of:     - The number of cases discussed.     - The types of patients and carers discussed.     - The types of patient and carer issues and problems discussed.     - The professionals present.     - The organisations represented.     - The decisions the meeting reached.     - The actions the meeting decided to take.     - Which professionals or organisations were tasked with carrying out actions.   - Reasons for atypicality.   - Other comments. - Part 4 – Completed by fieldwork researcher, post hoc.   - Reflections on:     - Professionals/agencies present at the meeting.     - How cases were introduced.     - How information was exchanged between participants.     - Which professionals contributed to discussions.     - Types of issues and problems the meetings sought to address.     - How decisions were reached.     - Who the key decision-makers were.     - Barriers identified.     - The overall ‘climate’ or ‘atmosphere’ of the meeting.     - Reflections on ‘added value’ of meetings.     - Other comments. |
